# Characteristics of the isocitrate dehydrogenase gene and telomerase reverse transcriptase promoter mutations in gliomas in Chinese patients

**DOI:** 10.1002/brb3.1583

**Published:** 2020-03-08

**Authors:** Chong‐Xiao Qu, Hong‐Ming Ji, Xiang‐Cheng Shi, Hong Bi, Li‐Qin Zhai, De‐Wu Han

**Affiliations:** ^1^ Department of Pathology Shanxi Provincial People's Hospital Taiyuan China; ^2^ Department of Pathophysiology Basic Medical Science Shanxi Medical University Taiyuan China; ^3^ Department of Neurosurgery Shanxi Provincial People's Hospital Taiyuan China

**Keywords:** Chinese gliomas, IDH mutation, mutation frequencies, overall survival analysis, sanger sequencing, TERT promoter mutation

## Abstract

**Objectives:**

To explore the characteristics of IDH and TERT promoter mutations in gliomas in Chinese patients.

**Methods:**

A total of 124 Chinese patients with gliomas were enrolled to study the frequencies of mutations in isocitrate dehydrogenase (IDH) and telomerase reverse transcriptase promoter (TERTp). Among the 124 patients, 59 patients were enrolled to study the classification of gliomas based on mutations in IDH and TERTp.

**Results:**

Isocitrate dehydrogenase mutations are positively correlated with a good prognosis but mutations in TERTp cannot predict prognoses independently. The combined analysis of the mutations of IDH and TERTp can predict the prognosis more accurately. Patients with IDH and TERTp glioma mutations have the best prognosis, followed by only IDH mutation patients and only TERTp mutation patients, which have the worst prognosis. IDH and TERTp mutations occur frequently in males, younger patients or lower‐grade patients. In contrast, only TERTp mutations occur frequently in females, older patients or higher‐grade patients.

**Conclusions:**

Patients with IDH and TERTp glioma mutations have the best prognosis, and only IDH mutation patients and only TERTp mutation patients have the worst prognosis. Moreover, the molecular classification of gliomas by mutations of IDH and TERTp is not suitable for pediatric patients.

## Graphical abstract

Patients with IDH and TERTp glioma mutations have the best prognosis, and only IDH mutation patients and only TERTp mutation patients have the worst prognosis. Both IDH and TERTp mutations occur frequently in males, younger patients, and lower‐grade patients. In contrast, only TERTp mutations occur frequently in females, older patients, and higher‐grade patients. Moreover, the molecular classification of gliomas by mutations of IDH and TERTp is not suitable for pediatric patients.

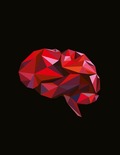

## 1 INTRODUCTION

Gliomas are the most common form of brain tumors and are classified as grade I to grade IV based on the histopathological and clinical criteria established by the World Health Organization (WHO) (Louis, Ohgaki, Wiestler, & Cavenee, 2016). Diffuse low‐grade and intermediate‐grade gliomas together make up the lower‐grade gliomas (WHO grades II and III), and glioblastoma was called higher‐grade gliomas (WHO grade IV) (Otani, Uzuka, & Ueki, 2017). Although there is long history of histopathological classification of gliomas, it does not adequately predict clinical outcomes due to the high intra‐observer and interobserver variability of this classification. Clinicians rely on genetic classifications to guide clinical decision‐making, especially for lower‐grade gliomas (Otani et al., 2017). For the first time, molecular parameters were introduced to define tumor entities together with histology in the 2016 WHO classification of tumors of the central nervous system (2016 CNS WHO) (Louis et al., 2016). Mutations in the isocitrate dehydrogenase gene (IDH, including IDH1 and IDH2) were reported to characterize the majority of lower‐grade gliomas and to be associated with better overall survival (OS) (Parsons et al., 2008). Mutations of telomerase reverse transcriptase promoter (TERTp) were subsequently found to frequently occur in gliomas (Lee et al., (2017)) and to be associated with a better outcome in the IDH mutation subgroup but a poorer outcome in the IDH wild‐type subgroup (Pekmezci et al., 2017). IDH and TERTp mutation status were often included in the studies of molecular classification of gliomas and to some extent, showed the ability to supplement a histopathological classification (Kim et al., 2018).

In this study, we explored IDH1, IDH2, and TERTp mutation status in a cohort of patients with gliomas from the WHO grade II to IV to identify the frequencies of these mutations in Chinese patients with gliomas.

## 2 MATERIALS AND METHODS

### 2.1 Patients and samples

Tumor tissue samples for genetic testing were taken from 124 patients who underwent surgeries at the Shanxi Provincial People's Hospital from April 2017 to November 2017. The ethics committee of the Shanxi Provincial People's Hospital approved the study. Written informed consent was obtained from all participants in the study.

### 2.2 DNA extraction

We performed DNA extraction from serial thick sections cut from the tumor tissue samples and control sections. Pathologists evaluated the invasive tumor content to ensure more than 50% of the cells were tumor cells. The DNA was isolated from the FFPE using the HiPure FFPE DNA Kit (D3126, Magen).

### 2.3 IDH1/IDH2 and TERT mutation analysis

Sanger sequencing (Shanghai Tongshu Biotechnology Co., Ltd) was used to determine the frequency of mutations in IDH1, IDH2, and TERTp (Sanger, Nicklen, & Coulson, 1997). The primer design was based on sequence data from accession numbers NM 005896 for IDH1, NM 002168 for IDH2, and NM 198253 for TERTp (http://www.ncbi.nlm.nih.gov). PCR amplification and Sanger sequencing were performed using an ABI‐PRISM 3730 DNA Analyzer (Applied Biosystems). PCR was performed in 20 μL reaction conditions including 20 ng of DNA. PCR amplification consisted of 16 cycles with denaturation at 95°C for 30 s, followed by annealing at 60°C for 30 s, and extension at 72°C for 30 s; thereafter, 25 cycles with denaturation at 95°C for 30 s, followed by annealing at 53°C for 30 s, and extension at 72°C for 30 s. Two microliters of the PCR amplification product was sequenced using the BigDye Terminator v3.1 Cycle Sequencing Kit (Applied Biosystems). Twenty‐five cycles were performed using the sense primers IDH1f 5′‐M13‐AAGTCACCAAGGATGCTGC‐3′, IDH2f 5′‐GCTGAAGAAGATGTGGAAAAGT‐3′, or TERTpf 5′‐AGCACCTCGCGGTAGTGG‐3′ with denaturation at 96°C for 10 s, annealing at 50°C for 5 s, and extension at 60°C for 4 min.

### 2.4 Statistical analysis

SPSS 25.0 was used for statistical analysis. Permutation test was used to compare the clinical characteristics between the different groups. *p* < .05 was considered statistically significant. Kaplan–Meier curves of OS were plotted for the TERTp mutation only group, IDH mutation only group, IDH and TERTp wild‐type group, and IDH and TERTp mutations group.

## 3 RESULTS

### 3.1 Demographic data of the patients

Among the 124 patients, only 67 patients had detailed clinical data. In the cohort, 40 were male, 27 were female, and the patients' ages ranged from 5 to 78 years. There were 12 oligodendrogliomas, 24 astrocytomas, 3 ependymomas, 2 gangliogliomas, and 26 glioblastomas. According to 2016 CNS WHO, the patients were classified as grade I (8), grade II (22), grade III (11), and grade IV (26) gliomas (1). Eight WHO grade I gliomas were excluded from further study for the reason that WHO grade I gliomas are often considered to be benign and are clinically and pathologically distinct from others; therefore, a final total of 59 patients were enrolled in this study. The baseline characteristics and clinicopathological information of the 59 patients were summarized in Table 1. All patients were treated with standard therapy.

**Table 1:** Clinical data of patients with IDH1 mutations, IDH2 mutations, and TERTp mutations in gliomas

	Total	IDH1 mutations			IDH2 mutations			TERTp mutations		
		+ (%)	− (%)	*p*	+ (%)	− (%)	*p*	+ (%)	− (%)	*p*
Gender										
Female	24 (40.7)	7 (11.9)	17 (28.8)	.585	0 (0)	24 (40.7)	1.000	19 (32.2)	5 (8.5)	.252
Male	35 (59.3)	13 (22.0)	22 (37.3)		1 (1.7)	34 (57.6)		22 (37.3)	13 (22.0)	
Age										
Mean age + *SD*	47.0 ± 16.2	45.8 ± 10.4	47.6 ± 18.4		48.0 ± 0	47.0 ± 16.3		49.0 ± 11.7	46.7 ± 16.7	
Median age	48.0	43.5	54.0		48.0	48.5		54.0	47.5	
≥48	30 (50.8)	6 (10.2)	24 (40.7)	.029	1 (1.7)	29 (49.2)	1.000	23 (39.0)	7 (11.9)	.267
<48	29 (49.2)	14 (23.7)	15 (25.4)		0 (0)	29 (49.2)		18 (30.5)	11 (18.6)	
Grade										
Lower grade (II‐III)	33 (55.9)	17 (28.8)	16 (27.1)	.002	1 (1.7)	32 (54.2)	1.000	21 (35.6)	12 (20.3)	.394
Higher grade (IV)	26 (44.1)	3 (5.1)	23 (39.0)		0 (0)	26 (44.1)		20 (33.9)	6 (10.2)	
Histologic type										
Oligodendroglioma	12 (20.3)	8 (13.6)	4 (6.8)		0 (0)	12 (20.3)		9 (15.3)	3 (5.1)	
Astrocytoma	19 (32.2)	8 (13.6)	11 (18.6)		1 (1.7)	18 (30.5)		12 (20.3)	7 (11.9)	
Glioblastoma	26 (44.1)	3 (5.1)	23 (39.0)		0 (0)	26 (44.1)		20 (33.9)	6 (10.2)	
Other	2 (3.4)	1 (1.7)	1 (1.7)		0	2 (3.4)		0	2 (3.4)	

Abbreviations: IDH, isocitrate dehydrogenase gene; TERTp, telomerase reverse transcriptase promoter.

### 3.2 Gene analysis results

Among the 124 gliomas, 35.5% (44/124) had a IDH1 mutation, of which 97.7% (43/44) was R132H, and 2.3% (1/44) was R132S; and 2.4% (3/124) had an IDH2 mutation, of which 2 cases had a mutation at site 172 (R172W, R172K), and 1 case had a mutation at site 174 (A174Y). Among them, 63.7% (79/124) had a TERTp mutation, of which 93.7% (74/79) was G228A, and 6.3% (5/79) was G250A.

Among the 59 gliomas, 40.7% (24/59) were female and 59.3% (35/59) were male; 50.8% (30/59) were older than 48 years and 49.2% (29/59) were younger than 48 years; and 55.9% (33/59) were lower‐grade (grade II or III) and 44.1% (26/59) were higher‐grade (grade IV). The mean age and median age were 47.0 and 48.0 years, respectively. Among the 59 gliomas, 33.9% (20/59) had an IDH1 mutation and 66.1% (39/59) were wild‐type IDH1. IDH1 mutations showed significant differences between the groups of different ages (*p* = .029) and pathological grades (*p* = .001), but there was no significant difference in gender (*p* = .585). Only 1.7% (1/59) had an IDH2 mutation, and 98.3% (58/59) were wild‐type IDH2. There were no significant differences in the distribution of IDH2 mutations between the groups of different gender (*p* = 1.000), ages (*p* = 1.000), or pathological grades (*p* = 1.000). Moreover, 69.5% (41/59) had a TERTp mutation and 30.5% (18/59) were wild‐type TERTp. There were no significant differences in the distribution of TERTp mutations between the groups of different gender (*p* = .252), ages (*p* = .267), or pathological grades (*p* = .394). The Kaplan–Meier estimates of OS in the gliomas with IDH mutations and TERTp mutations were shown in Figure 2a,b.

**Figure 1:**
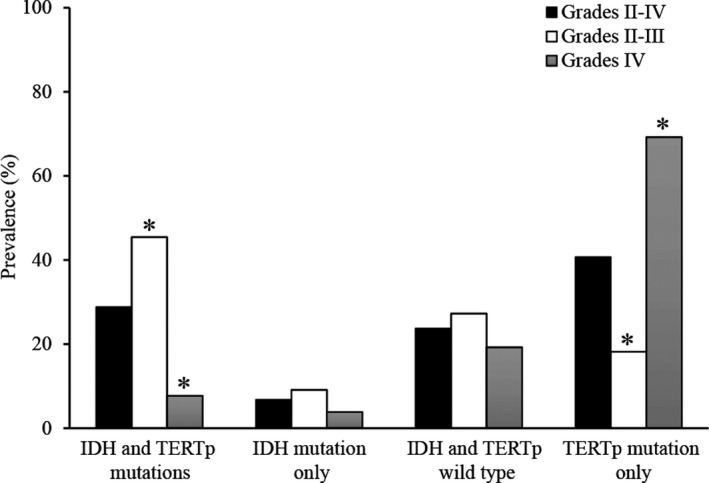
Prevalence of the glioma molecular groups in different grades. Bars Grades II‐IV, Grades II‐III, and Grades IV represent overall view, lower grade, and higher grade, respectively. * The distribution of different molecular groups between lower grade and higher grade was statistically significant

Based on the presence or absence of the two tumor markers, these gliomas were divided into four groups: IDH and TERTp mutations (17/59, 28.8%), IDH mutations only (4/59, 6.8%), IDH and TERTp wild type (14/59, 23.7%), and TERTp mutations only (24/59, 40.7%) (Table 2, Figure 1). For gliomas with mutations in both IDH and TERTp, the distribution between pathological grades (*p* = .001) was statistically significant, but there were no significant differences between the groups of different gender (*p* = .262) or age (*p* = .128). There was only 6.8% (4/59) of gliomas with only IDH mutations, and there were no significant differences between the groups of different gender (*p* = .694), age (*p* = .284), or pathological grades (*p* = .426). For gliomas with wild‐type IDH and TERTp, there were no significant differences between the groups of different gender (*p* = .291), age (*p* = .493), or pathological grades (*p* = .471). For gliomas with only TERTp mutations, the distributions between gender (*p* = .022), age (*p* = .011), and pathological grades (*p* < .001) were statistically significant.

**Table 2:** Relationship between clinical data with molecular groups in gliomas

	IDH and TERTp mutations No. (%)	IDH mutation only No. (%)	IDH and TERTp wild type No. (%)	TERTp mutation only No. (%)
Total	17 (28.8)	4 (6.8)	14 (23.7)	24 (40.7)
Gender				
Female	5 (29.4)	2 (50.0)	4 (28.6)	14 (58.3)
Male	12 (70.6)	2 (50.0)	10 (71.4)	10 (41.7)
Age				
Mean age + *SD*	41.0 ± 10.6	38.7 ± 19.8	29.3 ± 18.2	55.0 ± 8.4
Median age	41.0	48	17.0	58.0
≥48	6 (35.3)	1 (25.0)	6 (42.9)	17 (70.8)
<48	11 (64.7)	3 (75.0)	8 (57.1)	7 (29.2)
Grade				
Lower grade (II–III)	15 (88.2)	3 (75.0)	9 (64.3)	6 (25.0)
Higher grade (IV)	2 (11.8)	1 (25.0)	5 (35.7)	18 (75.0)
Histologic type				
Oligodendroglioma	7 (41.2)	1 (25.0)	3 (21.4)	2 (8.3)
Astrocytoma	8 (47.1)	1 (25.0)	5 (35.7)	4 (16.7)
Glioblastoma	2 (11.8)	1 (25.0)	5 (35.7)	18 (75.0)
Other	0	1 (25.0)	1 (7.1)	0

Abbreviations: IDH, isocitrate dehydrogenase gene; TERTp, telomerase reverse transcriptase promoter.

Among the 33 lower‐grade gliomas, 45.5% (15/33) had mutations in both IDH and TERTp, 9.1% (3/33) had mutations in IDH only, 27.3% (9/33) were wild‐type IDH and TERTp, and 18.2% (6/33) had mutations in TERTp only (Figure 1). The group with IDH and TERTp mutations and the group with wild‐type IDH and TERTp contained a large proportion of lower‐grade gliomas. Among the 26 higher‐grade gliomas, 7.7% (2/26) had mutations in both IDH and TERTp, 3.8% (1/26) had mutations in IDH only, 19.2% (5/26) were wild‐type IDH and TERTp, and 69.2% (18/26) had mutations in TERTp only (Figure 1). The group with only TERTp mutations dominated in higher‐grade gliomas. Although 75.0% (18/24) of gliomas with only TERTp mutations were higher‐grade gliomas, this group also contained 25.0% (6/24) lower‐grade gliomas (Table 2).

The mean age and median age at diagnosis were 41 years in the group with IDH and TERTp mutations, 38.7 and 48 years in the group with only IDH mutations, 29.3 and 17 years in the group with IDH and TERTp wild type, and 55 and 58 years in the group with only TERTp mutations, respectively. Patients who had gliomas with only TERTp mutations were older than the other patients (Table 2).

**Figure 2:**
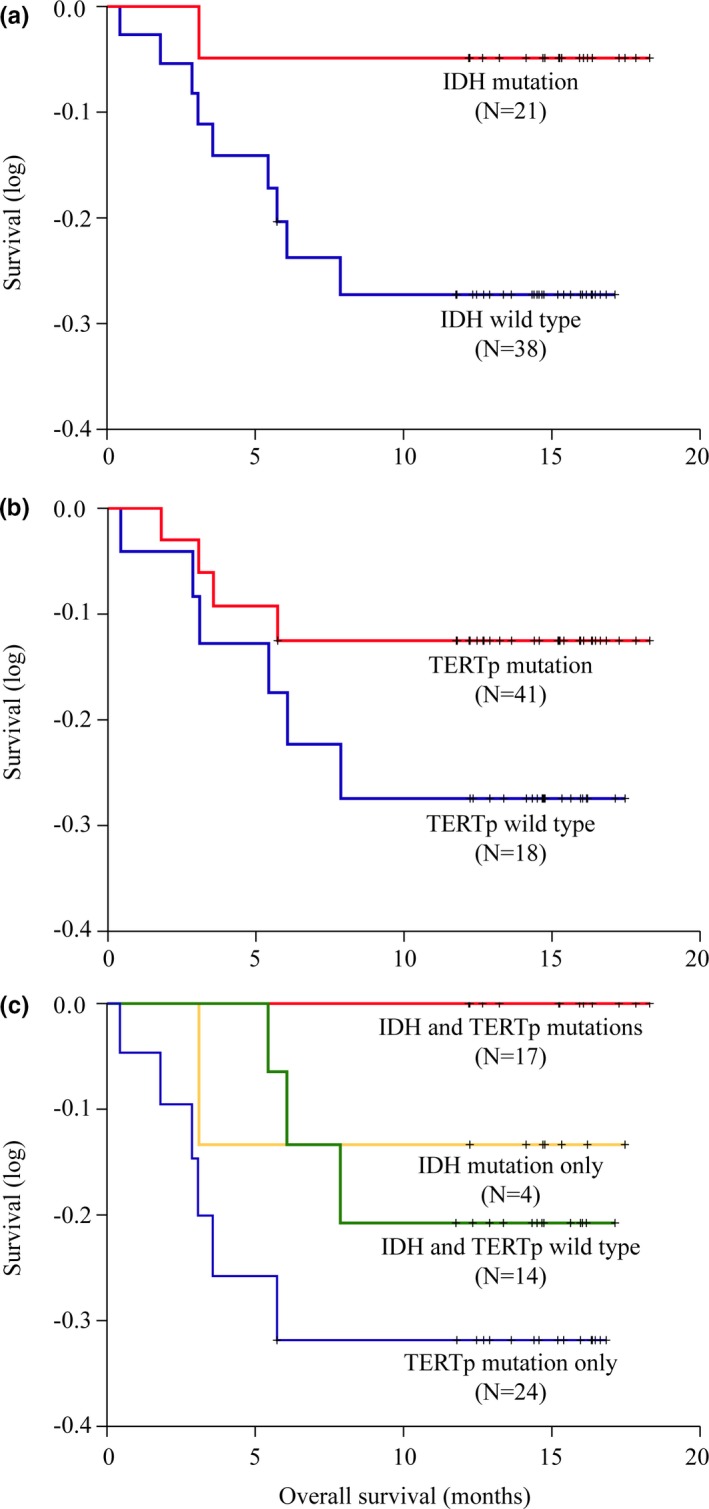
Kaplan–Meier estimates of overall survival of the gliomas with (a) IDH mutations; (b) TERTp mutations; (c) molecular classification based on IDH and TERTp mutation

The Kaplan–Meier estimates of OS in the glioma molecular groups were shown in Figure 2c. Patients who had gliomas with both IDH and TERT mutations had the best OS, and those who had gliomas with only TERTp mutations had the poorest OS.

## 4 DISCUSSION

Gliomas with IDH mutations have been reported to have a good prognosis (3) and our results also revealed a positive correlation between IDH mutation and a better OS in the Chinese population. IDH mutations occur in 80% of clinically diagnosed grade II and III gliomas but are rare in grade IV (Andronesi et al., 2012; Turcan et al., 2012). In our study, among the gliomas with IDH mutations, the lower‐grade (grade II or III) gliomas accounted for 85.7% (18/21) and the higher‐grade (grade IV) gliomas accounted for 14.3% (3/21). About 95% of all IDH mutations are in IDH1, and among those, over 90% are type R132H and less common mutants such as R132C, R132G, R132S, and R132L have also been identified (Balss et al., 2008; Hartmann et al., 2009; Ichimura et al., 2009; Pusch et al., 2014). In our study, mutations in R132H and R132S were 97.7% and 2.3%, respectively. IDH2 mutations rarely occur in gliomas and are commonly limited to IDH1 mutations (Dunn, Andronesi, & Cahill, 2013). In our study, only 2.4% (3/124) of the gliomas had IDH2 mutations. The vast majority of IDH mutated gliomas occur in patients younger than 55 years (Parsons et al., 2008; Robinson & Kleinschmidt‐DeMasters, 2017). In our study, only 9.5% (2/21) of patients with IDH mutated gliomas were older than 55 years. The incidence of IDH mutations in males was twice that in females, but the results did not show statistical significance.

The frequency of TERTp mutations is very high in gliomas (~50%), especially in glioblastomas (>60%) (Vinagre et al., 2013). Several studies have proposed that mutations in TERTp in glioblastomas are independently associated with a poor prognosis (Malkki, 2014; Simon et al., 2015). However, in studies involving different gliomas, Pekmezci et al. (2017) revealed that the wild‐type TERTp group was associated with a good prognosis only in wild‐type IDH1/IDH2 and the TERTp and IDH co‐altered group also had a better prognosis than found in the IDH mutation group. In our study, when we only considered the relationship between OS and TERTp mutation status, the wild‐type TERTp group showed worse prognosis than the TERTp mutation group, indicating that TERTp could not predict the prognosis independently (Figure 2b). TERTp mutations occur in 70%–83% of glioblastomas, 74%–78% of oligodendrogliomas, 25%–50% of oligoastrocytomas, and 10%–25% of astrocytomas (Pekmezci et al., 2017). Our results showed that TERTp mutations occurred in 76.9% of glioblastomas, 75% of oligodendrogliomas, and 63.2% of astrocytomas. Moreover, in our study, neither IDH mutations nor TERTp mutations occurred in pediatric gliomas. Previous studies also reported a low incidence of IDH mutations or TERTp mutations in pediatric gliomas: 16.3% and 0.5%, respectively (Koelsche et al., 2013; Pollack et al., 2011). This result indicates that IDH or TERTp mutations are not characteristic in pediatric patients with gliomas.

The IDH and TERTp mutation status is often taken into consideration in molecular classification studies of gliomas (Kim et al., 2018; Pekmezci et al., 2017). Patients with IDH and TERTp glioma mutations have the best prognosis whereas only IDH mutation patients and only TERTp mutation patients have the worst prognosis (Robinson & Kleinschmidt‐DeMasters, 2017). Our study showed similar results. Both IDH and TERTp mutations dominated in lower‐grade gliomas, and only TERTp mutations dominated in higher‐grade gliomas (Figure 1), which is consistent with the better prognosis of lower‐grade gliomas and the worse prognosis of higher‐grade gliomas. Gliomas with only TERTp mutations are primarily graded IV; however, 6 of the 24 (25%) in our study were grade II or III. Grade II or III gliomas in this group may have an aggressive course and may be associated with poor survival, which suggests the need for early adjuvant therapies and meticulous follow‐up sessions. Most gliomas with both IDH and TERTp mutations were presented in males, and those with only TERTp mutations were presented in females, indicating that male glioma patients usually have a better prognosis than females (Table 2). Most patients with gliomas with both IDH and TERTp mutations were younger than 48 years, and the majority of patients with only TERTp mutation gliomas were older than 48 years. The median age at diagnosis was the lowest (17 years) among patients who had gliomas with wild‐type IDH and TERTp and was highest (58 years) among the patients who had gliomas with only TERTp mutations. It is further suggested that molecular classification by IDH and TERTp is not suitable for pediatric patients with gliomas. Moreover, gliomas with only IDH mutations were generally at low frequency, younger than 48 years, and in the lower grade.

## 5 CONCLUSIONS

The results in the Chinese population are consistent with those in previous studies suggesting that patients with gliomas with IDH and TERTp mutations have the best prognosis whereas only IDH mutation patients and only TERTp mutation patients have the worst prognosis. Both IDH and TERTp mutations occur frequently in males, younger patients, and lower‐grade patients. In contrast, only TERTp mutations occur frequently in females, older patients, and higher‐grade patients. Moreover, the molecular classification of gliomas by mutations of IDH and TERTp is not suitable for pediatric patients.

## CONFLICT OF INTEREST

The authors declare that they have no conflict of interest.

## AUTHORS' CONTRIBUTIONS

CXQ, HMG, and XCS acquired data. CXQ, HMG, and DWH drafted the manuscript. DWH, HB, LQZ, and XCS contributed substantially to its revision. All authors take responsibility for the paper as a whole. All authors read and approved the final manuscript.

## ETHICS APPROVAL AND CONSENT TO PARTICIPATE

I confirm that I have read the Editorial Policy pages. This study was conducted with approval from the Ethics Committee of Shanxi Provincial People's Hospital. This study was conducted in accordance with the declaration of Helsinki.

## Data Availability

We declared that materials described in the manuscript, including all relevant raw data, will be freely available to any scientist wishing to use them for noncommercial purposes, without breaching participant confidentiality.
